# Shift of Feeding Strategies from Grazing to Different Forage Feeds Reshapes the Rumen Microbiota To Improve the Ability of Tibetan Sheep (Ovis aries) To Adapt to the Cold Season

**DOI:** 10.1128/spectrum.02816-22

**Published:** 2023-02-21

**Authors:** Xiongxiong Cui, Zhaofeng Wang, Penghui Guo, Fuhou Li, Shenghua Chang, Tianhai Yan, Huiru Zheng, Fujiang Hou

**Affiliations:** a State Key Laboratory of Herbage Improvement and Grassland Agro-ecosystems, Key Laboratory of Grassland Livestock Industry Innovation, Ministry of Agriculture and Rural Affairs, College of Pastoral Agriculture Science and Technology, Lanzhou University, Lanzhou, China; b Livestock Production Science Branch, Agri-Food and Biosciences Institute, Hillsborough, County Down, United Kingdom; c School of Computing, University of Ulster, Belfast, United Kingdom; Nanjing Agricultural University

**Keywords:** Tibetan sheep, feeding strategy, grazing, microbiota, cold season, Qinghai-Tibetan Plateau

## Abstract

The dynamics of ruminant-rumen microbiome symbiosis associated with feeding strategies in the cold season were examined. Twelve pure-grazing adult Tibetan sheep (Ovis aries) (18 months old; body weight, 40 ± 0.23 kg) were transferred from natural pasture to two indoor feedlots and fed either a native-pasture diet (NPF group) or an oat hay diet (OHF group) (*n* = 6 per treatment), and then the flexibility of rumen microbiomes to adapt to these compositionally different feeding strategies was examined. Principal-coordinate analysis and similarity analysis indicated that the rumen bacterial composition correlated with altered feeding strategies. Microbial diversity was higher in the grazing group than in those fed with native pasture and an oat hay diet (*P < *0.05). The dominant microbial phyla were *Bacteroidetes* and *Firmicutes*, and the core bacterial taxa comprised mostly (42.49% of shared operational taxonomic units [OTUs]) *Ruminococcaceae* (408 taxa), *Lachnospiraceae* (333 taxa), and *Prevotellaceae* (195 taxa), which were relatively stable across different treatments. Greater relative abundances of *Tenericutes* at the phylum level, *Pseudomonadales* at the order level, *Mollicutes* at the class level, and Pseudomonas at the genus level were observed in a grazing period than in the other two treatments (NPF and OHF) (*P* < 0.05). In the OHF group, due to the high nutritional quality of the forage, Tibetan sheep can produce high concentrations of short-chain fatty acids (SCFAs) and NH_3_-N by increasing the relative abundances of key bacteria in the rumen, such as *Lentisphaerae*, *Negativicutes*, *Selenomonadales*, *Veillonellaceae*, *Ruminococcus* 2, *Quinella*, *Bacteroidales* RF16 group, and *Prevotella* 1, to aid in nutrients degradation and energy utilization. The levels of beneficial bacteria were increased by the oat hay diet; these microbiotas are likely to help improve and maintain host health and metabolic ability in Tibetan sheep to adapt to cold environments. The rumen fermentation parameters were significantly influenced by feeding strategy in the cold season (*P < *0.05). Overall, the results of this study demonstrate the strong effect of feeding strategies on the rumen microbiota of Tibetan sheep, which provided a new idea for the nutrition regulation of Tibetan sheep grazing in the cold season on the Qinghai-Tibetan Plateau.

**IMPORTANCE** During the cold season, like other high-altitude mammals, Tibetan sheep have to adapt their physiological and nutritional strategies, as well as the structure and function of their rumen microbial community, to the seasonal variation of lower food availability and quality. This study focused on the changes and adaptability in the rumen microbiota of Tibetan sheep when they adapted from grazing to a high-efficiency feeding strategy during the cold season by analyzing the rumen microbiota of Tibetan sheep raised under the different management systems, and it shows the linkages among the rumen core and pan-bacteriomes, nutrient utilization, and rumen short-chain fatty acids. The findings from this study suggest that the feeding strategies potentially contribute to variations in the pan-rumen bacteriome, together with the core bacteriome. Fundamental knowledge on the rumen microbiomes and their roles in nutrient utilization furthers our understanding of how rumen microbial adaptation to harsh environments may function in hosts. The facts obtained from the present trial clarified the possible mechanisms of the positive effects of feeding strategy on nutrient utilization and rumen fermentation in harsh environments.

## INTRODUCTION

The alpine meadows of the Qinghai-Tibetan Plateau (QTP) constitute the largest area of pastoral grassland in China. Together, they are also the country’s largest, highest, and harshest-climate (cold, hypoxic) ecosystem and therefore pose a challenge to the survival of animals (1, 2). It has been documented that many native mammals are well adapted to this extreme environment (3–5). Nevertheless, host-microbiota relationships have garnered increased research attention in recent years, because evidence is accumulating that the gastrointestinal microbiotas have a close mutualistic symbiotic relationship with their hosts during long-term coevolution (6, 7). The unique microbiota of high-altitude ruminants enables them to stay healthy and survive in harsh environments. Many factors, such as host, lifestyle, season, diet, and age, influence the composition and function of rumen microbiota (8–10). The combination of these factors and the resulting microbiomes may be critical, as the health and behavior of a metaorganism are closely linked to the composition and function of its associated microorganisms, which may be forced to adapt whenever a change in the environment occurs (11).

Tibetan sheep (Ovis aries) is an indigenous breed at altitudes above 3,000 m on the QTP, an important part of China’s livestock industry, and the most important livestock species for the nomadic and seminomadic people who rely on it for their daily needs (e.g., milk, meat, fuel, skins, and wool) and income (12). Under traditional grazing management, Tibetan sheep mainly live on the native herbage of alpine meadow without feed supplementation. This herbage is inadequate during the long cold season, resulting in poor nutrition, health-related problems, and live-weight loss (13). However, despite these harsh environmental conditions, Tibetan sheep are well adapted and have thrived for thousands of years, maintaining their population (14). It has been reported that these animals possess greater regulation capacity in short-chain fatty acid (SCFA) metabolism pathways than small-tailed Han sheep, and this difference represents an advantage of Tibetan sheep over ordinary sheep in coping with low-energy intake (15). Nevertheless, during the cold season, like other high-altitude mammals, Tibetan sheep have to adapt their physiological and nutritional strategies, as well as the structure and function of their gastrointestinal tract microbial community, to the seasonal variation of lower food availability and quality (5, 16).

Rangeland has suffered from serious degradation for the past several decades as a consequence of frequent and strong anthropogenic activity, the grassland animal husbandry production faces great challenges (17). With the implementation of the government policy to “retire livestock and restore pasture,” oat (Avena sativa) has become a major forage supplement for the cold season (November to the following May) on the QTP owing to its cold tolerance, strong stress resistance, and high-yield characteristics (18, 19). Moreover, local herders have adopted management strategies to improve production efficiency, with livestock usually grazed on native pasture during the peak grass period or warm season, followed by the use of warm-shed feeding during the cold season (20). Under this production system, the growth and development of livestock can be improved effectively (21).

Due to the establishment of artificial grassland, during the warm-shed feeding period, besides dried native-pasture hay, local pastoralists can also choose oat hay as the main supplementary forage grass. Given that when grazing, Tibetan sheep ingest a high-variety diet, as they encounter different habitats and a variety of food types in the pasture, this can cause adaptive responses of the intestinal microbiota. As such, feeding strategy choice may provide a unique opportunity to manipulate the complex microbial ecosystem (22). Studies have demonstrated distinct rumen fermentation patterns of sheep switched from natural grazing to indoor feed depending on the feeding strategies (i.e., semigrazing with supplementation or barn feeding) (23). This difference in feeding strategies resulted in major changes in the nutrients ingested, especially fiber, water-soluble carbohydrates, and starch, and this also affected ruminants’ feed intake and gut microbiota.

Mounting evidence indicates that rumen microbiotas play a critical role in orchestrating nutrient absorption and metabolic homeostasis in Tibetan sheep (15). Tibetan sheep degraded fermentable substrates more efficiently when fed oat hay, as characterized by increased molar proportions of butyrate and propionate due to a shift in rumen fermentation pathways and microbial populations (24). This provided evidence for a correlation between the microbial community and SCFA production.

Our previous studies have emphasized the importance of the ruminal microbiome for nutrient digestion in the cold season and identified a core set of microbiotas that regulate host-microorganism interactions between ruminants and feed that has broad implications for ruminant health (18, 25, 26). Given the importance of Tibetan sheep on the QTP, we hypothesized that different feeding strategies would result in differences in the sheep ruminal microbiota and SCFA production and predicted that the differences would allow Tibetan sheep to cope better with the cold season. To test this prediction, we formulated two different diets (a low-quality forage [native pasture] and a higher-nutrient forage [oat]) and focused on the changes and adaptability in the rumen microbiota of Tibetan sheep when adapting from grazing to a high-efficiency feeding strategy during the cold season. To explore how the composition and function of the rumen microbiota can adapt to extreme cold, we used 16S rRNA sequencing to understand the dynamics of the rumen microbiota during an adaptation period of 3 weeks and hopefully shed light on the differences in the rumen microbiome and the SCFA profiles with special reference to the winter diet condition, thereby providing a valuable framework for grazing management strategies in Tibetan sheep from the perspective of nutrition and regulation.

## RESULTS

### Core bacterial communities and diversity.

We analyzed the 16S rRNA gene to estimate the rumen bacterial composition in a total of 24 samples of Tibetan sheep. Paired-end DNA amplicon sequencing of the partial 16S rRNA genes yielded 1,985,308 (mean ± standard deviation [SD] of 158,825 ± 2,743 reads per sample) high-quality bacterial sequences. Rarefaction curves for observed bacterial operational taxonomic units (OTUs) reach the saturation platform, indicating that the sequencing effort was sufficient to contain most of the microbial information in the sample (see Fig. S2 in the supplemental material).

**FIG 1:**
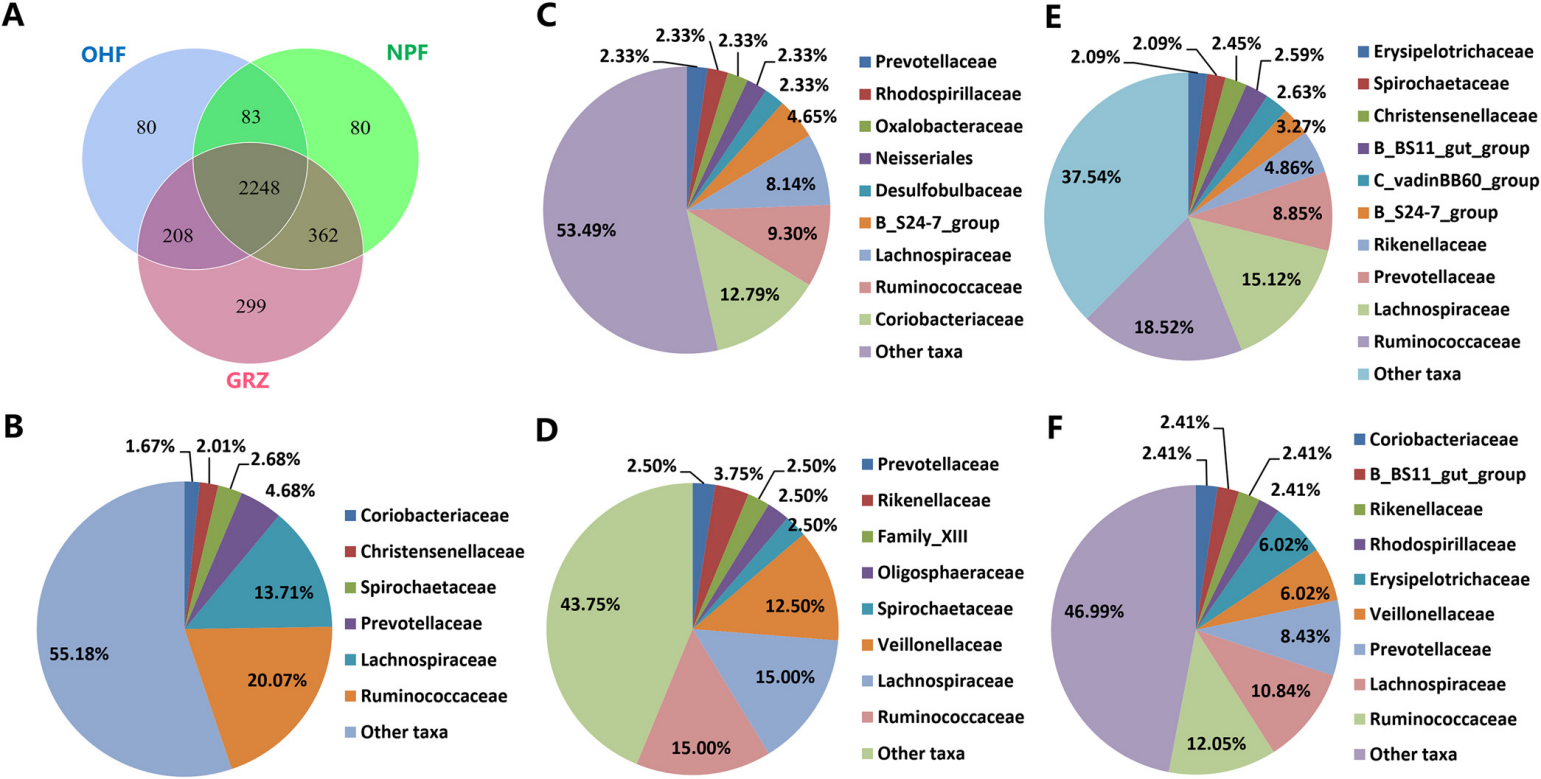
Shared and unique OTUs found in the rumen samples.

In all samples, a total of 3,360 OTUs were calculated, with the numbers of OTUs specific to the grazing (GRZ), native-pasture-fed (NPF), and oat-fed (OHF) groups being 299, 80, and 80, respectively (Fig. 1A). The annotation results of unique OTUs at the family level in the GRZ stage showed that these unique OTUs mainly belong to the families *Ruminococcaceae* (20.07%) and *Lachnospiraceae* (13.71%) (Fig. 1B). The unique bacterial families in the NPF group were mainly *Coriobacteriaceae* (12.79%), *Ruminococcaceae* (9.30%), and *Lachnospiraceae* (8.14%) (Fig. 1C); those for the OHF group were *Ruminococcaceae* (15%), *Lachnospiraceae* (15%), and *Veillonellaceae* (12.50%) (Fig. 1D). The number of OTUs shared among the three groups was 2,248 (representing 61.8%), and these can be considered to form part of the “core” ruminal bacteria of Tibetan sheep. The core OTUs identified belong to the phyla *Bacteroidetes* (13 families), *Firmicutes* (17 families), *Proteobacteria* (6 families), and other families belonging different phyla. The most abundant families were *Ruminococcaceae* (*Firmicutes*), *Lachnospiraceae* (*Firmicutes*), and *Prevotellaceae* (*Bacteroidetes*), with mean abundance values of 18.52%, 15.12%, and 8.85%, respectively (Fig. 1E). In the feeding stage, the 83 shared OTUs were mainly annotated to *Ruminococcaceae* (12.05%), *Lachnospiraceae* (10.84%), *Prevotellaceae* (8.43%), *Veillonellaceae* (6.02%), and *Erysipelotrichaceae* (6.02%) (Fig. 1F).

**FIG 2:**
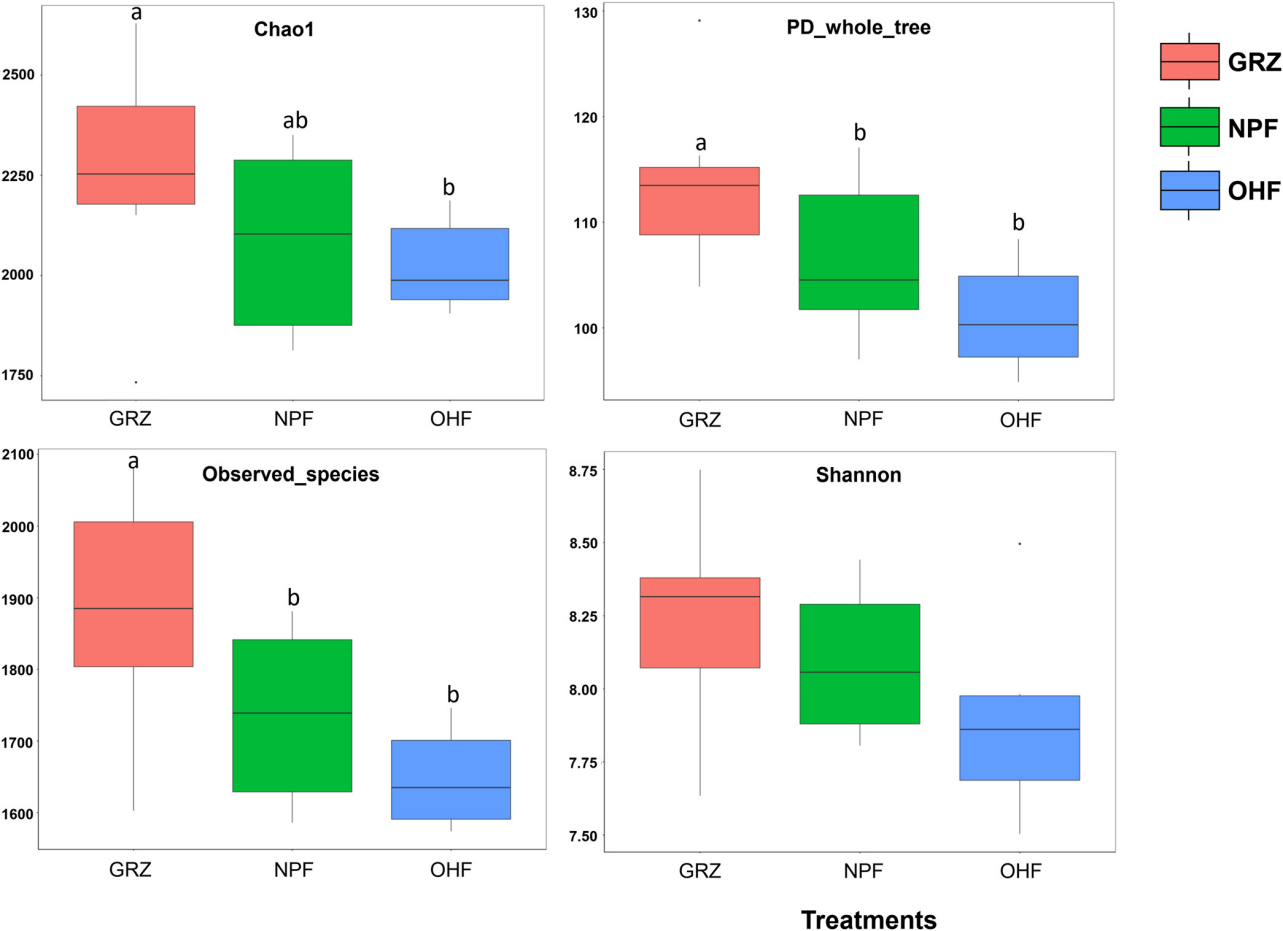
Box plot representation of alpha diversity indices in the Tibetan sheep rumen microbiome from the three treatment groups. GRZ, grazing group; NPF, native-pasture feed group; OHF, oat hay feed group.

The alpha diversity index analysis is shown in Fig. 2. Intergroup analysis of the community richness counts (Chao 1 estimator) intuitively showed that the abundance and diversity of the bacterial community in the GRZ group were higher than in the OHF group (*P < *0.05), but the difference between the NPF and OHF groups was not significant. The Shannon index is a reflection of homogeneity and richness of the community. The mean Shannon index of GRZ samples was slightly higher than those of the samples from the two experimental diets, i.e., NPF and OHF, and differences between diets with respect to Shannon index were not significant. The diversity parameters were the highest for GRZ samples, but differences between the two experimental groups (NPF and OHF) regarding diversity parameters were not significant.

### Rumen bacterial community composition.

**FIG 3:**
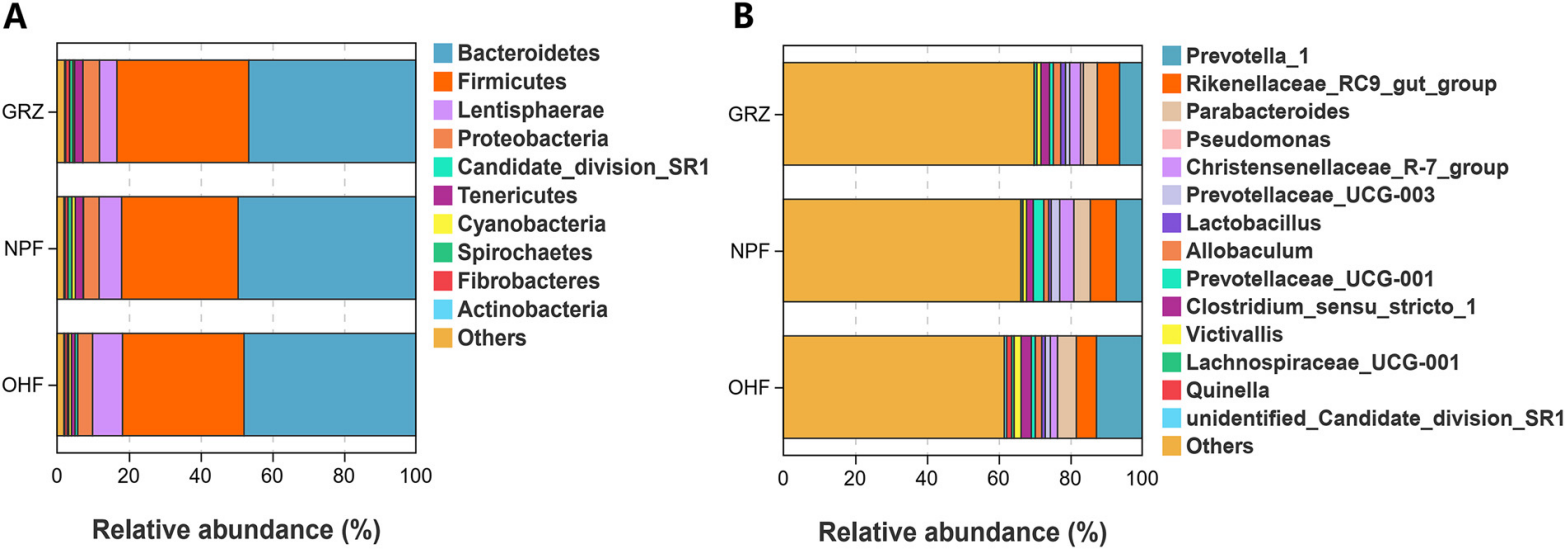
Classification of 16S rRNA gene sequences at the (A) phylum level for the grazing (GRZ) (*n *= 12), native-pasture-fed (NPF) (*n *= 6), and oat hay-fed (OHF) (*n *= 6) Tibetan sheep and (B) genus level in each group (present at ≥0.1% in at least one sample).

At the phylum level, 27 phyla were identified in the samples. Among these, *Bacteroidetes* (47.6%) and *Firmicutes* (35.0%) were detected as the dominant phyla regardless of group (Fig. 3A). In addition to these two phyla, the phyla *Lentisphaerae*, *Proteobacteria*, *candidate* division SR1, and *Tenericutes* had a relative abundance of ≥1% in all groups (6.1%, 4.4%, 2.5%, and 1.9%, respectively). At the family level, 130 families were identified in the rumen samples. *Prevotellaceae* (13.4%) and *Rikenellaceae* (10.6%) were detected as the dominant families regardless of group. At the genus level, the predominant members in the rumen of Tibetan sheep were *Prevotella* 1 (8.1%), *Rikenellaceae* RC9 gut group (6.3%), *Parabacteroides* (4.4%), and *Christensenellaceae* R-7 group (3.0%) (Fig. 3B). In order to provide clarity and visualization, a heat map showed the top 35 genera (Fig. S3).

**FIG 4:**
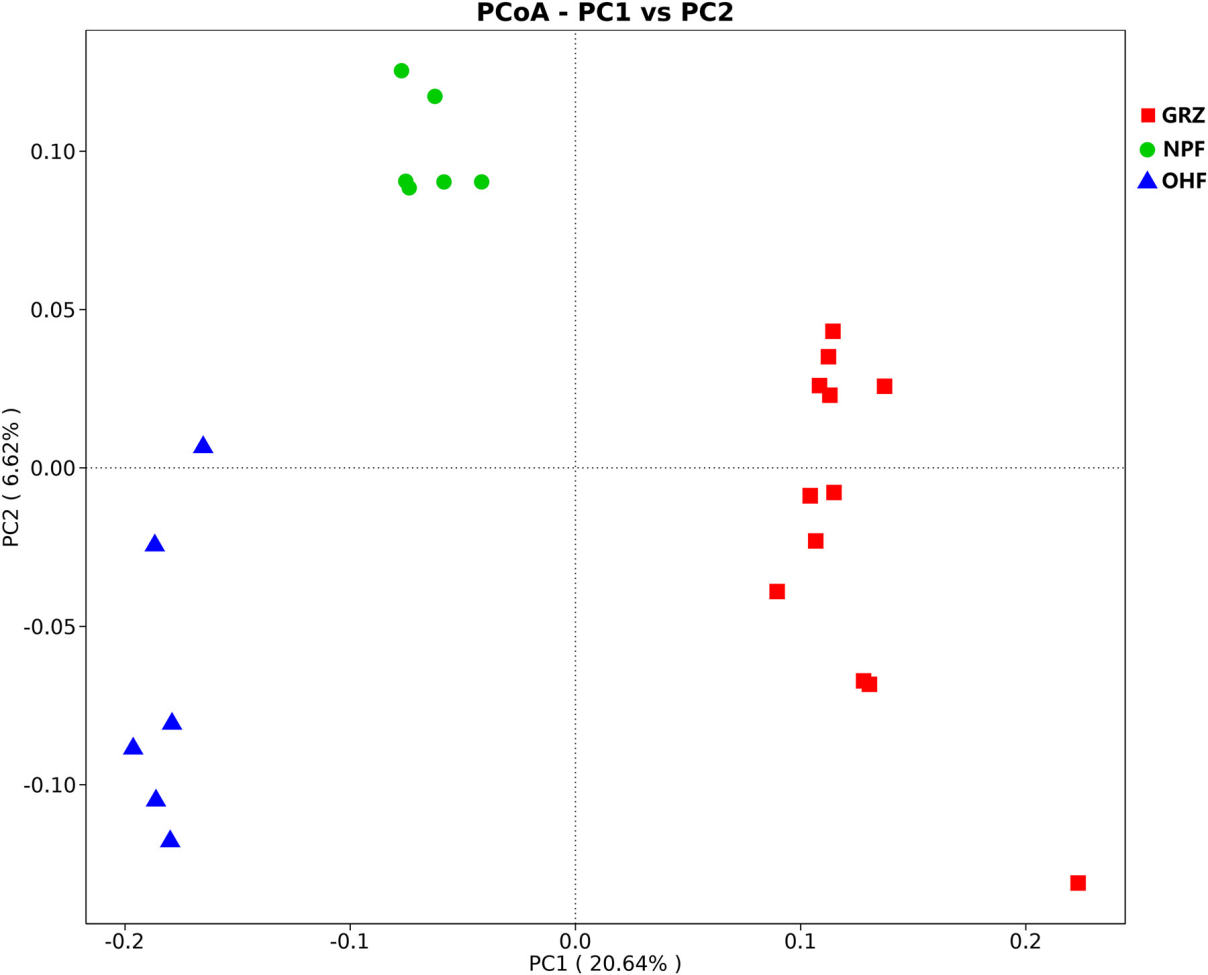
PCoA of the taxon distribution of all experimental samples.

According to the principal-coordinate analysis (PCoA) (explained variance = 20.64% and 6.62% for the first and second coordinates, respectively) based on the Jaccard distances (Fig. 4), all 24 microbial samples can be classified into three clearly separated clusters: the rumen fluid samples of Tibetan sheep from GRZ, the rumen fluid samples of the NPF group, and the rumen fluid samples of the OHF group. Interestingly, the OHF clusters showed higher intersample variability, and the rumen fluid microbiota samples were sequentially distributed along the second coordinate (i.e., the *y* axis).

Multivariate one-way analysis of variance (ANOVA) based on multiresponse permutation procedure (MRPP) was run to test the significance of diet, environmental variables, and time with regard to differences in rumen bacteria considering all samples (GRZ-NPF, 0.001; GRZ-OHF, 0.003; NPF-OHF, 0.002).

### LEfSe analysis of bacterial features under different treatments.

**FIG 5:**
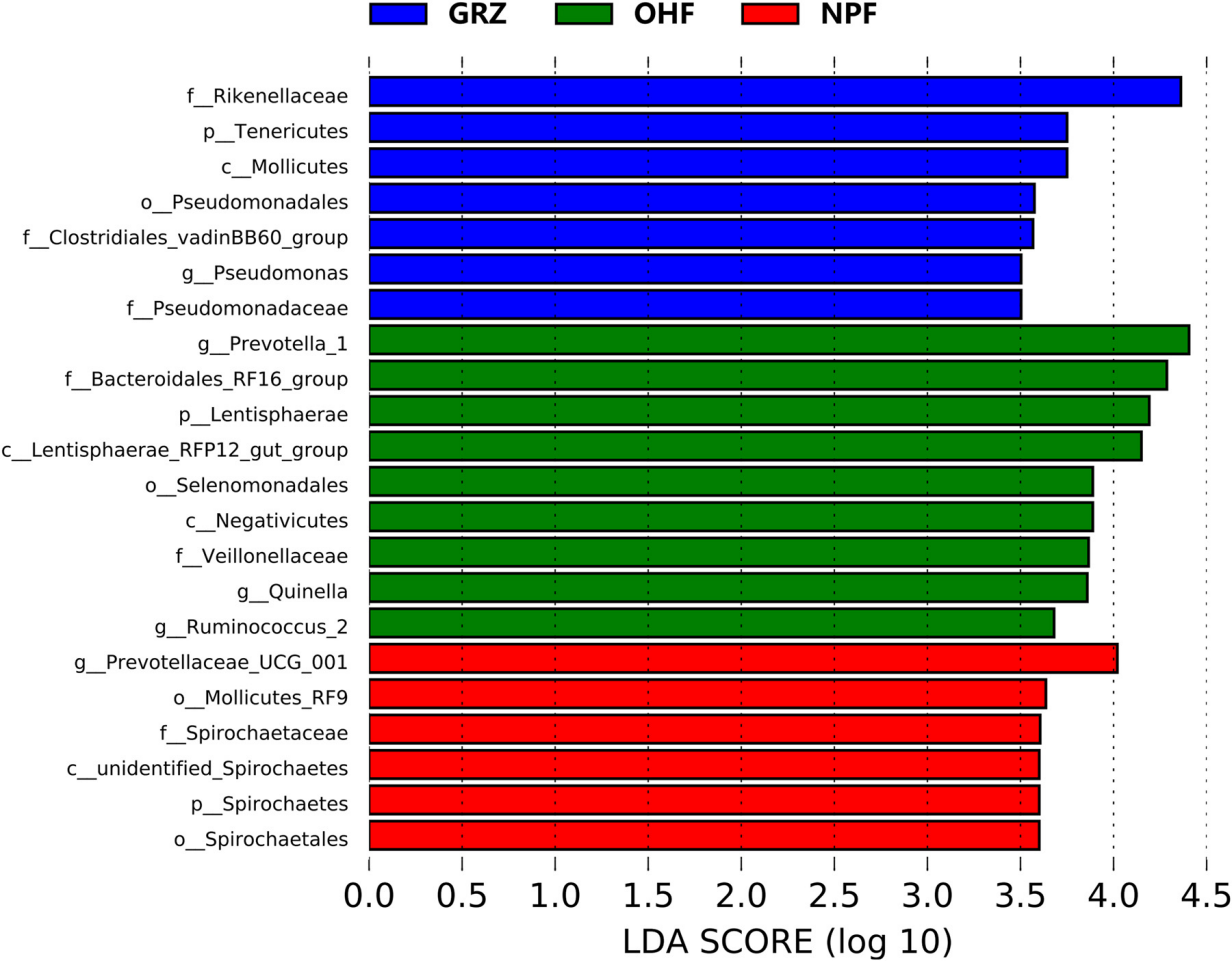
Identified biomarkers in rumen fluid samples of Tibetan sheep in three groups using LEfSe analysis (LDA score > 3.5 and *P < *0.05).

Based on the above analyses of similarity, diversity, and composition of the rumen microbiota, we classified the colonization of the rumen microbiota at two time points into three treatments: GRZ (day 0), NPF, and OHF. To determine the functional communities in samples, linear discriminant analysis (LDA) effect size (LEfSe) analysis (LDA score > 3.5 and *P* < 0.05) was conducted to identify the groups that displayed significant differences across the different treatments. A total of 22 unique bacterial biomarkers in the rumen fluid of Tibetan sheep among the three groups are shown in a histogram (Fig. 5). We identified nine bacterial taxa as biomarkers of the OHF group, including *Lentisphaerae* and its one member (i.e., *Lentisphaerae* RFP12 gut group), *Negativicutes*, *Selenomonadales*, *Veillonellaceae*, *Ruminococcus* 2, *Quinella*, *Bacteroidales* RF16 group, and *Prevotella* 1, and six bacterial taxa, including *Mollicutes* RF9, *Prevotellaceae* UCG 001, *Spirochaetes*, and its three members (i.e., unidentified *Spirochaetes*, *Spirochaetales*, and *Spirochaetaceae*), were detected as biomarkers in the rumen of NPF sheep. In addition, we found seven bacterial taxa that were more abundant in the GRZ group, including *Tenericutes* and its one member (i.e., *Mollicutes*), *Clostridiales* vadinBB60 group, *Rikenellaceae*, and three bacterial taxa (*Pseudomonadales*, *Pseudomonadaceae*, and Pseudomonas) belonging to the class *Gammaproteobacteria*. Clearly, there was a reconstruction within the rumen bacteria with the change in feeding strategy in the cold season.

### Rumen fermentation parameters.

There were no significant differences in the rumen pH, butyrate, isobutyrate, and valerate concentrations in Tibetan sheep between the three groups (*P < *0.05; Table 1). The concentrations of total volatile fatty acids (TVFA) and ammonia-nitrogen (NH_3_-N) were significantly influenced by feeding strategy and forage type (*P < *0.05). The proportions of NH_3_-N, propionate, and TVFA in the OHF group were higher than in the GRZ and NPF groups (*P < *0.05). The proportions of isovalerate, valerate, and acetate-to-propionate ratio in the OHF group were lower than in the GRZ and NPF groups (*P < *0.05).

**TABLE 1:** Rumen fermentation parameters of Tibetan sheep

Parameter^*a*^	Value for strategy^*b*^			SEM	*P*
	GRZ	NPF	OHF		
pH	6.74	6.67	6.63	0.05	0.627
NH_3_-N (mg/dL)	15.19a	18.26b	24.11c	0.93	0.001
TVFA (mmol/L)	29.13a	29.58a	46.35b	1.72	0.001
Molar proportion of VFA (%)					
Acetate	72.94a	68.93b	69.10b	0.64	0.004
Propionate	16.09a	17.62ab	18.85b	0.46	0.033
Isobutyrate	1.21	1.18	1.03	0.06	0.510
Butyrate	7.83	7.98	7.45	0.14	0.432
Isovalerate	1.07a	0.77b	0.52c	0.06	0.001
Valerate	0.78	0.65	0.50	0.28	0.108
A/P	4.67a	3.95ab	3.67b	0.17	0.024

a A/P, acetate/propionate ratio.

b Means in a row with different letters differ significantly (*P* < 0.05).

### Correlation analysis.

**FIG 6:**
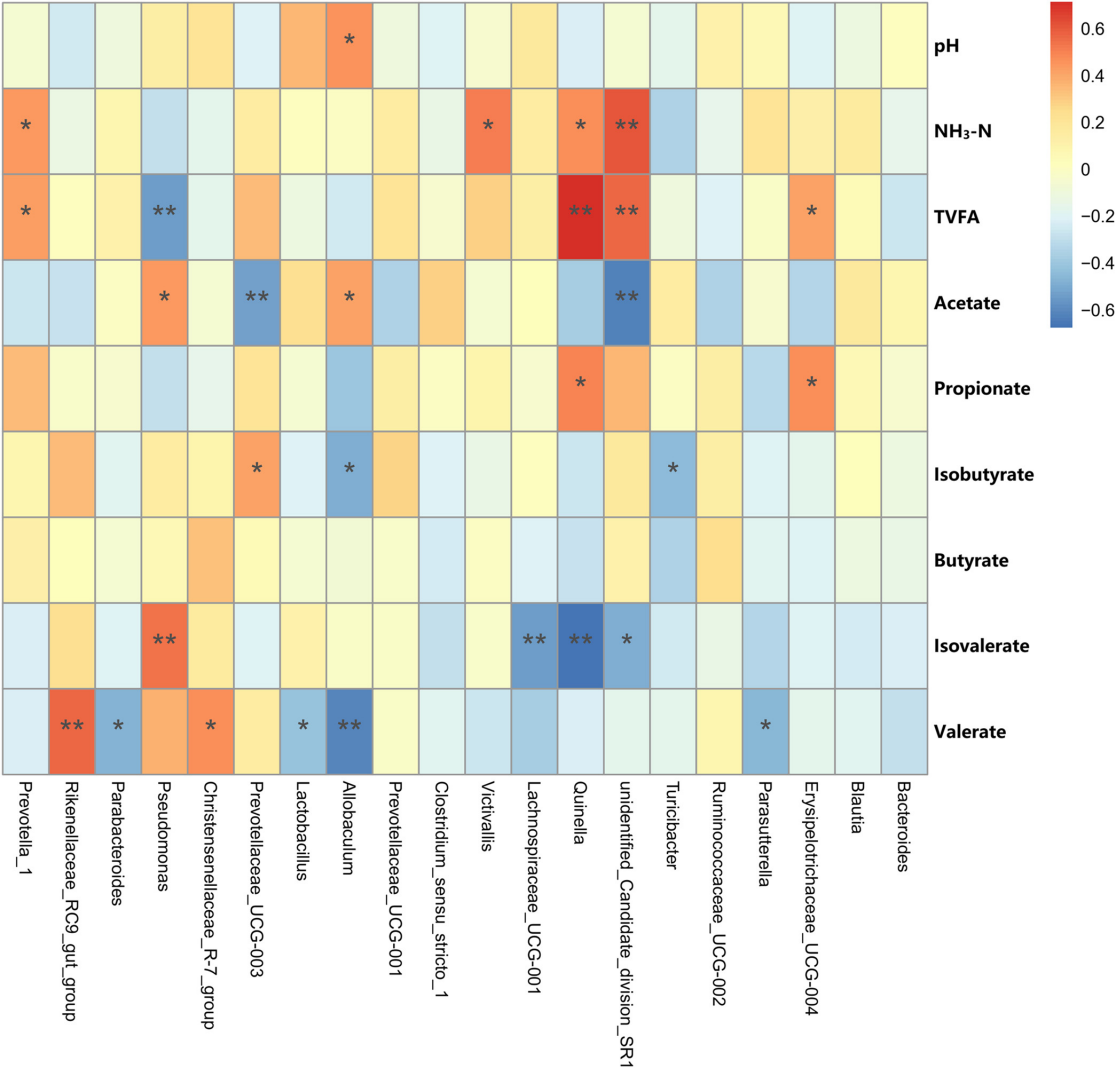
Relationships among pH, NH_3_-N, TVFA, SCFAs, and bacterial genera (present at ≥0.1% in at least one sample).

Correlation analysis was performed to identify the correlation between the fermentation parameters and the relative abundances of the rumen bacteria (Fig. 6). The TVFA and NH_3_-N concentrations and the abundances of the bacterial genera were closely related to each other (*P < *0.05). The rumen pH was positively correlated with the relative abundance of the genus *Allobaculum*. The NH_3_-N concentration was positively correlated with the relative abundances of the genera unidentified *candidate* division SR1, *Prevotella* 1, *Quinella*, and *Victivallis*. The acetate molar proportion was positively correlated with *Allobaculum* and *Parasutterella* and negatively correlated with the abundances of unidentified *Candidate* division SR1 and *Prevotellaceae* UCG-003. The propionate molar proportion was positively correlated with the relative abundances of the genera *Quinella* and *Erysipelotrichaceae* UCG-004. The isobutyrate molar proportion was positively correlated with the relative abundance of *Prevotellaceae* UCG-003 and negatively correlated with the abundances of *Allobaculum* and *Turicibacter*. The valerate molar proportion was positively associated with *Rikenellaceae* RC9 gut group and *Christensenellaceae* R-7 group and negatively correlated with the abundances of *Parasutterella*, *Lactobacillus*, and *Allobaculum*. The isovalerate molar proportion was positively associated with Pseudomonas and negatively correlated with the abundances of *Quinella*, *Lachnospiraceae* UCG-001, and unidentified *Candidate* division SR1. The TVFA concentration was directly associated with the relative abundances of the genera *Prevotella* 1, *Quinella*, unidentified *Candidate* division SR1, *Erysipelotrichaceae* UCG-004, and *Butyrivibrio* 2 and negatively correlated with Pseudomonas.

**FIG 7:**
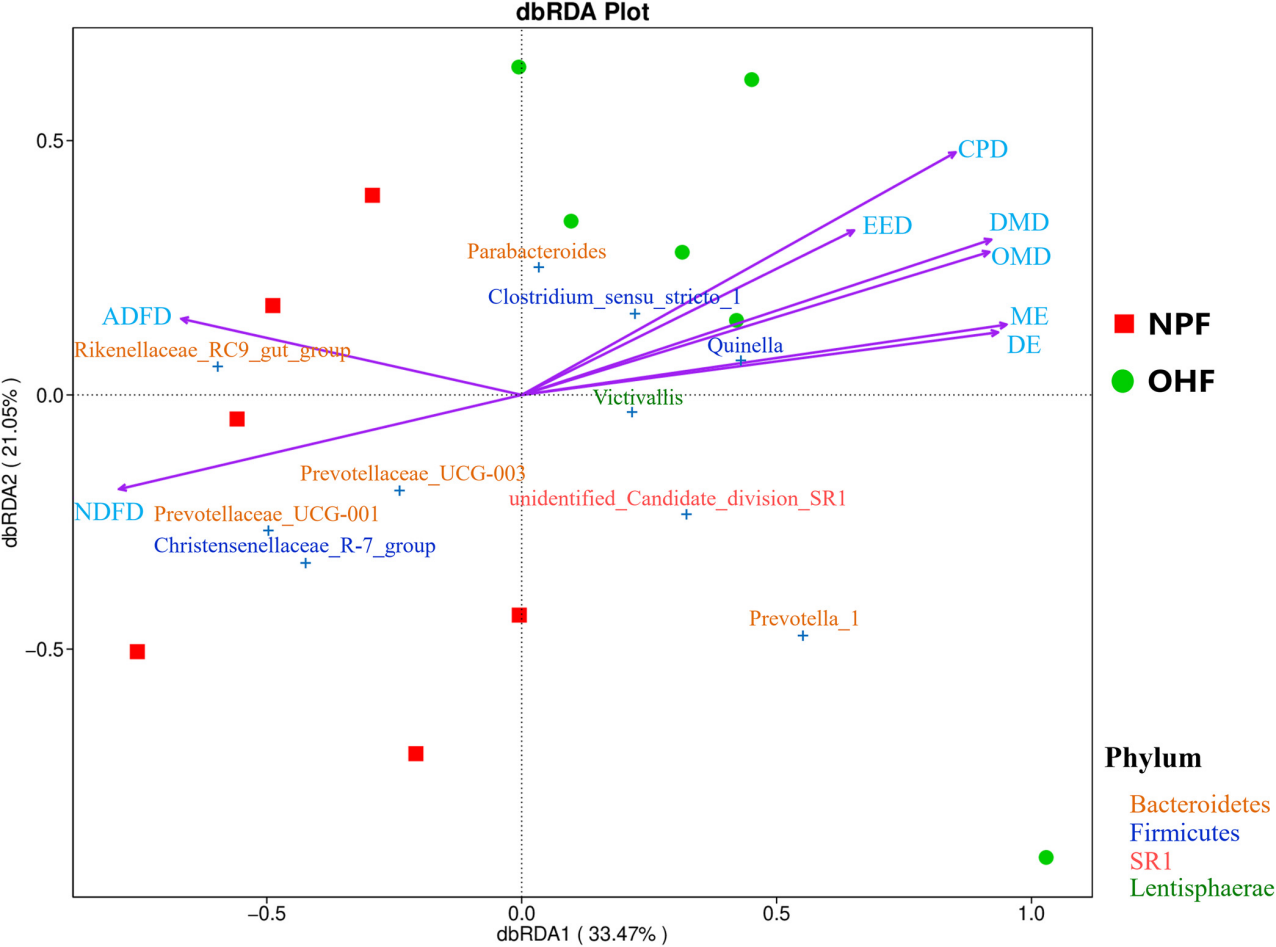
Biplot from the RDA that shows the relationships between the bacterial community composition at the genus level and the physiological status for two groups. Projecting a sample’s point at right angles on an arrow approximates the position of the sample along that variable, and the distance between two samples’ points approximates their difference in bacterial communities; the cosine values of the angles between explanatory variables reflect their correlations.

Eight physiological index factors (i.e., apparent digestibility of dry matter [DMD], apparent digestibility of organic matter [OMD], apparent digestibility of neutral detergent fiber [NDFD], apparent digestibility of acid detergent fiber [ADFD], apparent digestibility of ether extract [EED], apparent digestibility of crude protein [CPD], digestible energy [DE], and metabolizable energy [ME]) were found to be correlated with the bacterial communities at the genus level by redundancy analysis (RDA). Coloring the samples by their different diet groups showed that the centroids of the two groups’ clouds were well separated, which confirmed the remarkable correlation between the physiological indexes of the sheep and their rumen bacterial community (Fig. 7). By plotting the bacteria on the RDA map, we were able to find the bacteria most related to a specific variable. For example, *Quinella*, unidentified *Candidate* division SR1, and *Prevotella* 1, located in the positive direction of the DMD arrow, were positively related to DMD, whereas *Christensenellaceae* R-7 group was negatively related to DMD, OMD, and EED. More information was uncovered by comparing the angles between the arrows; for example, OMD, EED, CPD, ME, and DE were positively related to DMD, as their arrows expanded in almost the same direction, whereas ADFD and NDFD were negatively related to DMD. We found that *Prevotella* 1 and *Quinella*, which were more abundant in the OHF group, were strongly and positively correlated with DMD, OMD, ME, and DE (Fig. S4).

## DISCUSSION

In this study, high-throughput sequencing was used to assess the changes in rumen bacteria of Tibetan sheep fed two different diets during the feeding cycle. The current research demonstrates that the response of bacterial communities in the rumen of Tibetan sheep to feeding strategies can differ markedly during the overwintering stage, thereby facilitating adaptation to the challenging alterations in the harsh environment and available diet on the QTP.

Although rumen microbial community composition varies with host, diet, and other experimental treatments, a core microbiome remains stable regardless of the conditions. In this study, there were 2,076 OTUs across experimental parameters within the core bacteria; and sheep fed a native-pasture-based diet or oat hay-based diet were all found to have 80 unique OTUs. Gharechahi et al. (27) found 746 core OTUs in camels fed with woody shrubs and low-quality natural forage. Meanwhile, the core microbiome of dairy cows was found to consist of 26 genera and 82 OTUs in a study that utilized a high-quality silage-based diet (28). More recently, McCann et al. (29) observed 22 core OTUs in steers fed a high-quality Bermuda grass diet. In the present study, the predominant phyla in the rumen of Tibetan sheep were *Bacteroidetes* (47.6%) followed by *Firmicutes* (35.0%), which together constituted more than 82.60% of each sample. These findings are consistent with many past reports on the QTP (19, 26).

Whether ruminants are fed a concentrate or hay-based diet, *Firmicutes* and *Bacteroidetes* are ubiquitously distributed in the bacterial communities of their rumen, indicating their functional and ecological significance in the digestive tract. In the current study, *Ruminococcaceae* (*Firmicutes*), *Lachnospiraceae* (*Firmicutes*), and *Prevotellaceae* (*Bacteroidetes*) were the most abundant bacterial families, ubiquitous in all rumen samples. At the genus level, *Prevotella* 1, *Rikenellaceae* RC9 gut group, *Parabacteroides*, and *Christensenellaceae* R-7 group constituted both the main bacteria and core bacterial community, as reported previously (25).

*Prevotella* 1 is known for its considerable functional versatility and genetic divergence, and its species are the main participants in the efficient utilization of hemicelluloses, starch degradation, and protein peptide metabolism (30). *Rikenellaceae* RC9 gut group is the genus that plays the most important role in protein and primary or secondary carbohydrate degradation (31, 32). *Christensenellaceae* R-7 group has been speculated to be related to cellulose degradation activities and associated with health (33, 34). Although *Parabacteroides* members have been associated with cattle fed lower-forage diets (35), a description of their functions in the rumen is lacking. Despite the shifts in the rumen bacterial community composition at different levels, we identified 83 stable bacterial OTUs in two shed-feeding groups, among which 10 (12.05%) were taxonomically assigned to *Ruminococcaceae*, 9 (10.84%) were *Lachnospiraceae*, and 7 (8.43%) were *Prevotellaceae*, representing the most prevalent families in the rumen of shed-feeding sheep. These bacterial taxa have been suggested to be part of the core microbiome in the rumen (36). There is ample experimental evidence that multiple OTUs belonging to *Prevotellaceae*, *Ruminococcaceae*, and *Lachnospiraceae* are correlated with cold stress adaptation, energy storage, and gross feed efficiency in ruminants (5, 25, 26, 37), suggesting that the stable OTUs in ruminal bacteria we observed may possess conserved and important functional and ecological implications for the ruminant host.

Macroscopically, the bacterial richness of rumen significantly decreased in the group foraging on the oat hay/native-pasture diet in comparison to the GRZ group, and the shed-feeding diet also curtailed the within-habitat diversity of the rumen microbiota. The diversity of prokaryotes has previously been reported to be lower in total mixed-ration-fed ruminants compared to ruminants fed on pasture (38). The shed-feeding sheep in this study also had lower diversity of bacteria, indicating that rumen bacterial communities of native-pasture/oat hay-fed and mixed ration-fed ruminants may share some characteristics. Tibetan sheep graze mostly on *Gramineae*, *Cyperaceae*, *Leguminosae*, and other species. Harsh dietary conditions might influence the rumen microbiota to ferment and digest fibrous materials as a main dietary ingredient in order to maximize nutrient extraction (39).

Ordinarily, high species diversity provides more functional redundancy and is usually related to strong stability (40). The food sources accessible to grazing sheep are more diverse, thus providing sheep with many different nutrient types, which may mean that a more diversified rumen bacterial community is required to help Tibetan sheep fully utilize these nutrients (41). Additionally, social behavior has been shown to correlate with gut microbiota diversity, and the shed-feeding sheep in this study typically foraged independently in a separate space from the grazing sheep, which foraged in herds. Specifically, interactions with a greater number of individuals and frequent social interactions will promote species richness in individual gut microbiota communities (42). In summary, the high stability and diversity of the rumen microbial system of the Tibetan sheep probably reflect its generalist herbivore strategy.

Ruminal microbial composition is known to be closely associated with the utilization efficiency of forage, as a stable microbial composition plays a vital role in maintaining a good performance in producing animal products. Rumen microbial community structure is highly responsive to variation in diet and feeding strategy (32), as confirmed by analysis of similarity (ANOSIM) and 16S rRNA gene analysis. In a grazing environment, Tibetan sheep indiscriminately capture any food that they can obtain, and sometimes they prefer some common poisonous weeds, which are rich in secondary metabolites. In autumn, alpine meadows have entered the withering period. With the advance of the withering period, the quantity and quality of forage decrease sharply, especially in the low-temperature season, which is the most difficult period for Tibetan sheep to survive.

In our study, although the relative abundance of *Bacteroidetes* in the grazing group was lower than in other groups, some bacterial taxa of lower abundance, such as *Tenericutes*, *Clostridiales* vadinBB60 group, *Rikenellaceae*, and Pseudomonas, were significantly higher in the GRZ group than in those fed with native pasture and an oat hay diet. The apparent resilience of the diversity of this microbial community in the face of seasonal dietary change and underlying physiological stress is probably an effective adaptation for natural grazing ruminants. In the cold season, Tibetan sheep under grazing management may experience more pronounced nutrient and/or low-temperature stress, and the harsh living environment may have resulted in a relatively stable and diversified microbial community in its inhabitants. When Tibetan sheep migrate to indoor feedlots, they lose the possibility of an autonomous choice of feeding, and gastrointestinal tract microbiota changes are usual, as available forage alters spatially and nutritionally. These differences in the microbiota in the gut may be the result of differences in climate conditions, diet composition, stress exposure, and energy utilization (43). As Tibetan sheep have been subjected to shed feeding, they have had to develop strategies to cope with such a drastic change in lifestyle. These adaptations potentially contribute to rumen microbiota divergence among hosts from different dietary guilds (44).

In the present study, the phylum *Tenericutes* differed among the three groups, being enriched in the grazing group. This phylum has been described in previous studies and consists of four orders (*Anaeroplasmatales*, *Entomoplasmatales*, *Mycoplasmatales*, and *Acholeplasmatales*) belonging to the class *Mollicutes*, of which several members have been found to be animal parasites and pathogens (45). Moreover, the high-throughput sequencing data of this study revealed that the transition from grazing to a native-pasture diet resulted in a decreasing trend of *Firmicutes*. *Firmicutes* members play an important role in energy conversion, and the ratio of *Firmicutes* to *Bacteroidetes* in ruminants is strongly related to animal obesity and body fat storage (46, 47). The variation in the microbial biota, directly related to available forage and forage quality, has been recorded in other herbivores grazing on the grassland (2, 23). Under grazing conditions, Tibetan sheep need to consume their own energy to survive in a relatively harsh environment, while the barn environment is relatively comfortable and the range of activities is limited, thereby reducing energy consumption and helping to alleviate the phenomenon of fat loss. The decrease in the *Firmicutes*-to-*Bacteroidetes* ratio demonstrates that Tibetan sheep might exhibit improved suitability to winter cold stress.

The enrichment of many bacterial members in the rumen can help improve host resistance to the low-temperature environment in winter, as well as metabolic capacity (48). In this research, the oat hay diet increased the level of the phylum *Lentisphaerae*, and some members of this phylum have a potential role in cellobiose degradation (49). Moreover, correlation analysis has revealed that *Lentisphaerae* plays an important role in the microbial degradation of gut pentoses via the sedoheptulose-1,7-bisphosphate pathway (50). At the genus level, the bacterial taxa *Ruminococcus* 2, *Prevotella* 1, and *Quinella*, which are associated with propionate production, nutrient uptake and utilization, immune function, and protein and saturated fatty acid fermentation (51–53), were enriched in the OHF sheep. The members of *Prevotellaceae* have genetically and metabolically diverse microbial populations in the rumen that are widely considered to have the ability to efficiently degrade lignocellulosic feedstock, protein, and pectin (54). *Prevotella* 1 might utilize polysaccharides and simple sugars to produce propionate (55). *Ruminococcus* plays an important role in biodegradation of resistant starch and other dietary fiber (56). *Quinella* is associated with lower methane production in sheep and is an important propionate-producing bacterium (52). These rumen microbes allow Tibetan sheep to obtain energy through the utilization of food, whereas energy compensation strategies permit Tibetan sheep survival in harsh withering period environments.

Compared with natural herbage, the higher contents of soluble carbohydrate, biodegradation of resistant starch, EE, and CP, and the lower contents of ADF and NDF in the oat hay diet may be the reason for the differences in the relative abundance of these bacteria in the rumen. This suggests that feeding with oat hay leads to an increase in beneficial bacteria and better carbohydrate decomposition and maintenance of energy in the cold season. These genera are likely to help improve and maintain host health and the metabolic ability of Tibetan sheep to adapt to the cold and harsh environment of the QTP.

During the cold season, animals mobilize their body reserves to support energy utilization, which generally leads to loss of body mass. A certain level of insight into the positive effects of physiological and nutritional strategies in Tibetan sheep is provided here by the correlations that have been established between nutrient digestibility, the rumen fermentation parameters, and microbial profiles. The difference in carbohydrate and fiber composition in the forage leads to different VFA profiles, and high acetic acid production in the rumen is associated with a high-fiber diet (57).

In this study, acetate was the dominant SCFA in the rumen of Tibetan sheep, which may be a major energy source for host and rumen microbiotas (58); in addition, the values were higher in the grazing sheep than in the NPF and OHF groups, which could be due to higher fiber degradation (26). The decreased acetate production can be explained by the reduction of certain Gram-positive bacteria (59). The *Lachnospiraceae* and *Ruminococcaceae* are also known for producing acetate as main end products (60). In the present study, the decreased populations of *Lachnospiraceae* might also have contributed to the deterioration in acetate production in the oat hay diet. Propionic acid production is closely related to the NDF-to-starch ratio in the diet of ruminants (61). In the present study, we found that the increased concentrations of propionate and TVFA in sheep fed the oat hay diet, compared with native-pasture-feeding sheep, could have been due to the high content of nonfibrous carbohydrate in oat hay. Therefore, an oat hay diet might result in a specific rumen ecology that facilitates the fermentation of the rumen during the cold season.

Propionate is produced in the ruminal ecosystem by two major pathways (the succinate pathway and acrylate pathway), and a large number of microbes play important roles in these pathways (59, 62). Moreover, propionate is the most important VFA for net synthesis of glucose (63). Thus, an increase in propionate in the rumen is a positive sign of improved energy utilization, as well as the easing of cold stress. In the present study, *Quinella* and *Erysipelotrichaceae* UCG-004 were positively associated with the concentrations of propionate. However, because of the complexity of the interaction between bacteria, it is difficult to know which bacteria directly cause the production of a specific VFA (64).

NH_3_-N concentrations are associated with the rate of nitrogen use by rumen microorganisms. Some functional rumen bacteria, such as *Prevotella* and *Quinella*, can improve the digestibility of dietary protein and metabolism of amino acids, resulting in the production of high concentrations of NH_3_-N (65, 66). This result is consistent with the greater abundance of *Prevotella* and the higher concentrations of NH_3_-N in the Tibetan sheep of the OHF group. To summarize, the oat hay diet increases protein degradation in Tibetan sheep and might partially explain the increased digestibility of crude protein and ruminal NH_3_-N concentration. Therefore, we concluded that a higher abundance of functional bacteria in the rumen improves forage digestibility, while producing high concentrations of SCFAs and NH_3_-N to rapidly improve the growth performance of Tibetan sheep in the cold season.

### Conclusion.

In this preliminary study, the rumen bacterial flora of Tibetan sheep exposed to two different feeding regimens in the cold season were determined, and the interaction between Tibetan sheep rumen microbes and SCFAs of the host and their regulatory mechanisms were explored. Our results clearly identify large-scale variation in the ruminal microbial ecosystems of young Tibetan sheep in response to the physiochemical composition of grass-forage diets and feeding strategies. Upon transferring Tibetan sheep from pasture to indoor feeding, the relative abundance of microorganisms was significantly altered, and the beta diversity significantly differed too, while the alpha diversity was significantly reduced. The abundance of pathogenic bacteria that was higher in the grazing state was reduced in the two indoor feedlots, benefiting sheep health. In the OHF treatment, the high relative abundance of *Prevotella* 1 and *Quinella* promoted forage fermentation to produce a high concentration of TVFA and NH_3_-N, while some functional members mainly involved in cellulose decomposition, crude protein and ether extract digestion, and energy utilization were significantly enriched, potentially increasing plant biomass decomposition and improving the growth of Tibetan sheep. Collectively, this study furthers our understanding of how rumen microbial adaptation to harsh environments may function in hosts, providing insights into the tripartite relationship between the microbiota, diet, and environment, and the sampling scheme might be useful in future studies. Further research should focus on the relationship between rumen microbes, niche diet changes, and low-temperature habitats.

## MATERIALS AND METHODS

### Experimental design, dietary treatments, sampling, laboratory analysis, and calculation.

Feeding trials were conducted from December to the following January (i.e., the coldest season) at the Animal Husbandry Science and Technology Demonstration Park (35°58′N, 101°53′E; elevation, 3,700 m above sea level [ASL]), Maqu County, Gansu Province, China. The average relative humidity and temperature during the experimental period were 45% and −15.8°C.

A total of 12 adult Tibetan sheep (male; 18 months old) in good body condition (average initial body mass of 40 ± 0.23 kg) were selected from local pasture as test animals. On day 0, the animals were ear tagged, and rumen fluid samples were taken from each sheep at the start of the trial (baseline) (GRZ; *n *= 12). Then, sheep were randomly assigned to one of two dietary treatments: native-pasture feeding (NPF; *n *= 6) or oat hay fed (OHF; *n *= 6). The sheep were housed in individual pens (1 m by 1.2 m), were fed daily at 8:00, 12:00, 15:00, and 18:00, and had *ad libitum* access to diets and water. The native forage was cut each day from native alpine meadow pasture close to the research station, and oat hay was obtained from the cultivated pasture near the test station. Both native forage and oat harvested were chopped into approximately 2- to 5-cm lengths before being fed to sheep. Table 2 shows the feeding value of oat hay compared with native-pasture grass evaluated in this study. The total experimental period comprised 21 days, including a 15-day pretest and a 6-day formal test (Fig. S1).

**TABLE 2:** Feed quality indicators of diets

Feed quality indicator	Proportion in diet^*a*^		SEM	*P*
	Native pasture	Oat hay		
Dry matter	91.55	90.07	0.27	0.001
Ash	6.23	5.50	0.11	0.000
Crude protein	5.04	7.50	0.30	0.000
Neutral detergent fiber	67.93	54.13	2.13	0.000
Acid detergent fiber	39.37	31.17	1.25	0.000
Ether extract/crude fat	1.81	2.12	0.05	0.000
Gross energy	17.90	17.92	0.02	0.532

a Values are percentage of dry matter, except for gross energy, which is in megajoules per kilogram.

Rumen fluid samples (~50 mL) were collected 2 h after the morning feed using an oral stomach tube on day 21 of the experimental period. The sample pH was immediately measured using a digital 206 pH2 meter (Testo, Germany). All samples were immediately sealed in a centrifuge tube, transported to our laboratory within 24 h, and stored at −80°C in a medical refrigerator for subsequent analysis. All animal experimental procedures were reviewed and approved by the Ethics and Animal Welfare Committee of Lanzhou University (file no. 2012-1 and 2012-2).

A detailed description of the trial site, animal feeding, laboratory analyses, and calculation of physiological indexes (DMD, OMD, NDFD, ADFD, CPD, EED, DE, and ME) can be found in a complementary paper on Tibetan sheep (18). Therefore, only brief methodological descriptions are provided here. During the final 6 days, the feed intake of each sheep was measured daily, and samples of forage and feces were collected. The forage and feces samples DM, OM, CP (Kjeldahl N × 6.25), NDF, ADF, and EE content were determined as described previously (18): the total N content was measured by the Kjeldahl method; the gross energy was determined by bomb calorimetry (6400; Parr, Inc., Moline, IL, USA); the ADF and NDF were determined using an ANKOM 2000 fiber analyzer (ANKOM Technology, Fairport, NY, USA), and the EE was determined with an ANKOM XT-15 analyzer (ANKOM Technology, Macedon, NY, USA).

NH_3_-N concentrations was determined using a phenol-hypochlorite assay with a Cary 60 UV-Vis spectrophotometer (Agilent Technologies, Inc., Santa Clara, CA, USA) at an absorbance of 630 nm, as described by Broderick and Kang (67). The analysis of SCFA concentrations was carried out as described by Li et al. (68).

### DNA extraction, PCR amplification, and sequencing.

Rumen fluid samples (−78°C) were sent to Novogene Bioinformatics Technology (Beijing, China) for the determination of microorganisms. Each sample was thawed separately and centrifuged at 4°C, and then 200 μL of the supernatant was taken for further analysis. The cetyltrimethylammonium bromide (CTAB) method was used to extract total genomic DNA following the operating procedures (69). The concentration of total DNA from each sample was estimated through the *A*_260_/*A*_280_ ratio (1.8 to 2.2) by using a NanoDrop 2000 spectrophotometer (NanoDrop Technologies, Wilmington, DE, USA), and the purity and integrity of the extracted DNA were assessed using 1% agarose gel electrophoresis (Axygen Biosciences, Union City, CA, USA). Variable region 4 (V4) of the 16S rRNA gene present in these 24 rumen fluid samples were sequenced using a HiSeq PE250 sequencing platform (Illumina). The V4 hypervariable region of the bacterial 16S rRNA gene was prepared from each of the DNA samples using the primer set 515F/806R (515F, 5′-GTGCCAGCMGCCGCGGTAA-3′; reverse primer 806R, 5′-GGACTACHVGGGTWTCTAAT-3′) and Phusion R high-fidelity PCR master mix with GC buffer (New England Biolabs, Ipswich, MA, USA). Cycling parameters included initial denaturation at 95°C for 5 min, followed by 25 cycles at 95°C for 1 min, 55°C for 1 min, and 72°C for 2 min, with a final extension at 72°C for 8 min. The PCR products were detected by 2% agarose gel electrophoresis, purified using the Qiagen DNA gel extraction kit (Qiagen Biosciences, Qiagen, Hilden, Germany) according to the manufacturer’s instructions, and then quantified with a fluorometer.

The obtained 16S rRNA gene sequencing raw data were processed using QIIME pipeline version 1.8.0 (http://qiime.org). Quality filtering of the raw tags was done using FLASH (version 1.2.7; http://ccb.jhu.edu/software/FLASH/) with default parameters (70). Chimera sequences were identified and removed using the UCHIME algorithm (http://www.drive5.com/usearch/manual/uchime_algo.html) and Gold database (http://drive5.com/uchime/uchime_download.html) (71, 72). Effective tags were clustered by UPARSE 7.0.1001 (http://drive5.com/uparse/) into OTUs based on 97% similarity (73). Based on the SILVA database (74), the software MUSCLE (version 3.8.31; http://www.drive5.com/muscle/) was used to conduct and normalize the multiple sequence alignment.

### Data and statistical analysis.

Alpha diversity index analyses, including Chao 1 (species richness) index, Shannon index (species diversity), PD whole tree (phylogenetic diversity), and Sobs (observed species richness), were based on normalized data, calculated with QIIME 1.8.0, and displayed with R (version 2.15.3). The significant rankings of abundant modules in different groups were revealed using the LEfSe technique. Bacterial community structure was visualized by applying PCoA based on the binary Jaccard distances using R (version 2.15.3). Pearson correlation coefficients were used to assess the associations between ruminal bacteria (genus) and environmental factors. We related differences in bacterial composition to the physiological index factors of Tibetan sheep using redundancy analysis (RDA), as implemented in R (version 2.15.3).

All statistical analyses were analyzed with SPSS (version 25.0; IBM Corp., Armonk, NY, USA). The differences in chemical compositions of experimental diets and rumen fermentation parameters were analyzed using one-way ANOVA. The nonparametric Kruskal-Wallis test was used to compare the bacterial relative abundances and diversity between the three treatments using SPSS 25.0. *P* values of less than 0.05 were taken as statistically significant, and values of less than 0.01 were considered extremely statistically significant.

### Data availability.

The raw reads were deposited at NCBI (BioProject accession ID PRJNA903276).
