# Supplementary material for: Shift of Feeding Strategies from Grazing to Different Forage Feeds Reshapes the Rumen Microbiota To Improve the Ability of Tibetan Sheep (Ovis aries) To Adapt to the Cold Season
Source: Microbiol Spectr. 2023 Feb 21;11(2):e02816-22. doi: 10.1128/spectrum.02816-22 (PMC10100778; doi:10.1128/spectrum.02816-22)
Supplement: Supplemental file 1 — Supplemental material. Download spectrum.02816-22-s0001.pdf, PDF file, 0.4 MB [file spectrum.02816-22-s0001.pdf]

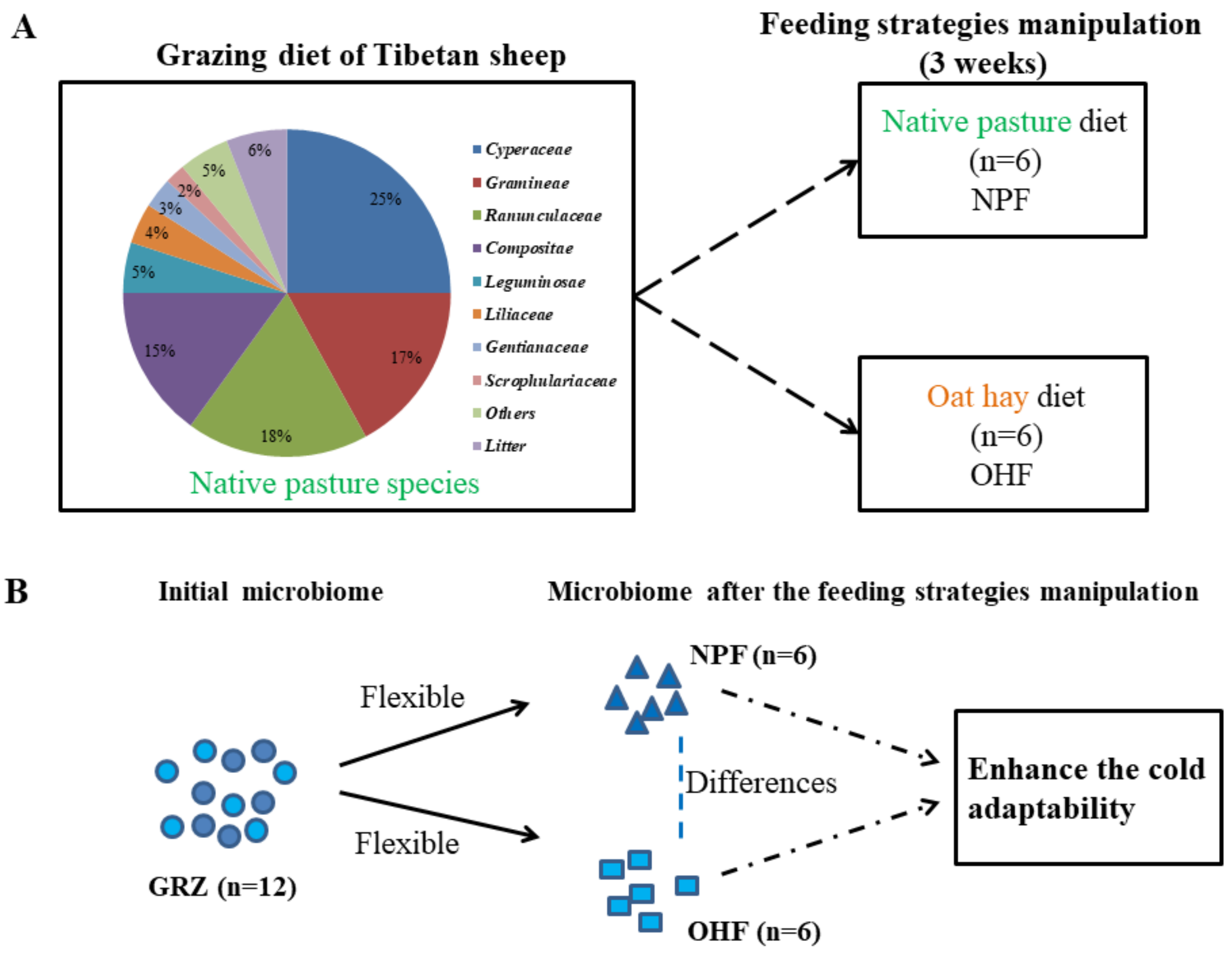

Supplementary Fig 1 (A) Contents of native pasture diet, with a schematic timeline of the experiment. (B) Predicted flexible (toward two diet groups) microbiome responses to dietary changes in Tibetan sheep in the cold season on the Qinghai-Tibetan Plateau. GRZ, grazing group; NPF, native pasture feed group; OHF, oat hay feed group.

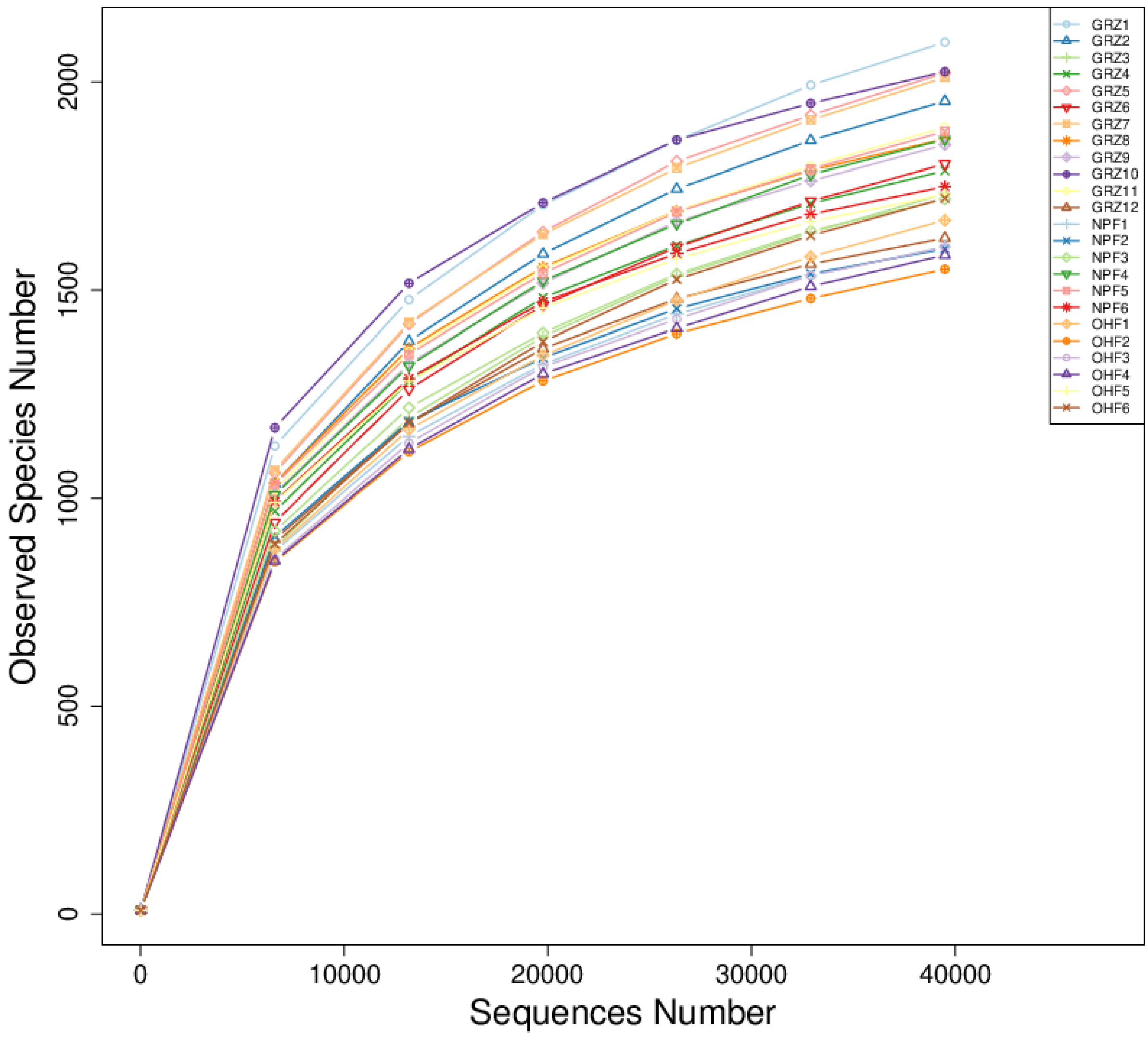

Supplementary Fig 2 Rarefaction analysis among the 24 different samples at the OUT level. GRZ, grazing group; NPF, native pasture feed group; OHF, oat hay feed group.

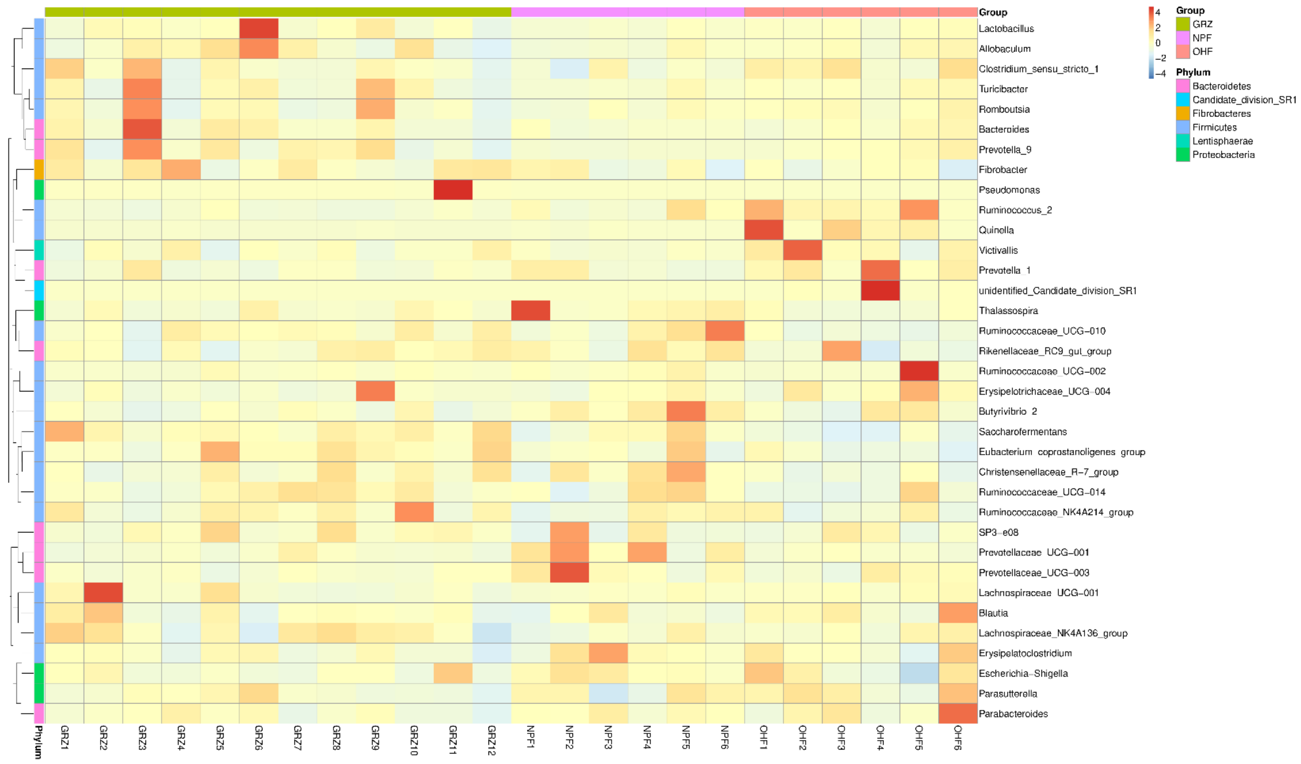

Supplementary Fig 3 Heatmap analysis of 35 shared prokaryotic community genera of all Tibetan sheep, as determined by the relative abundance of each shared genera through sequencing platform. GRZ, grazing group; NPF, native pasture feed group; OHF, oat hay feed group.

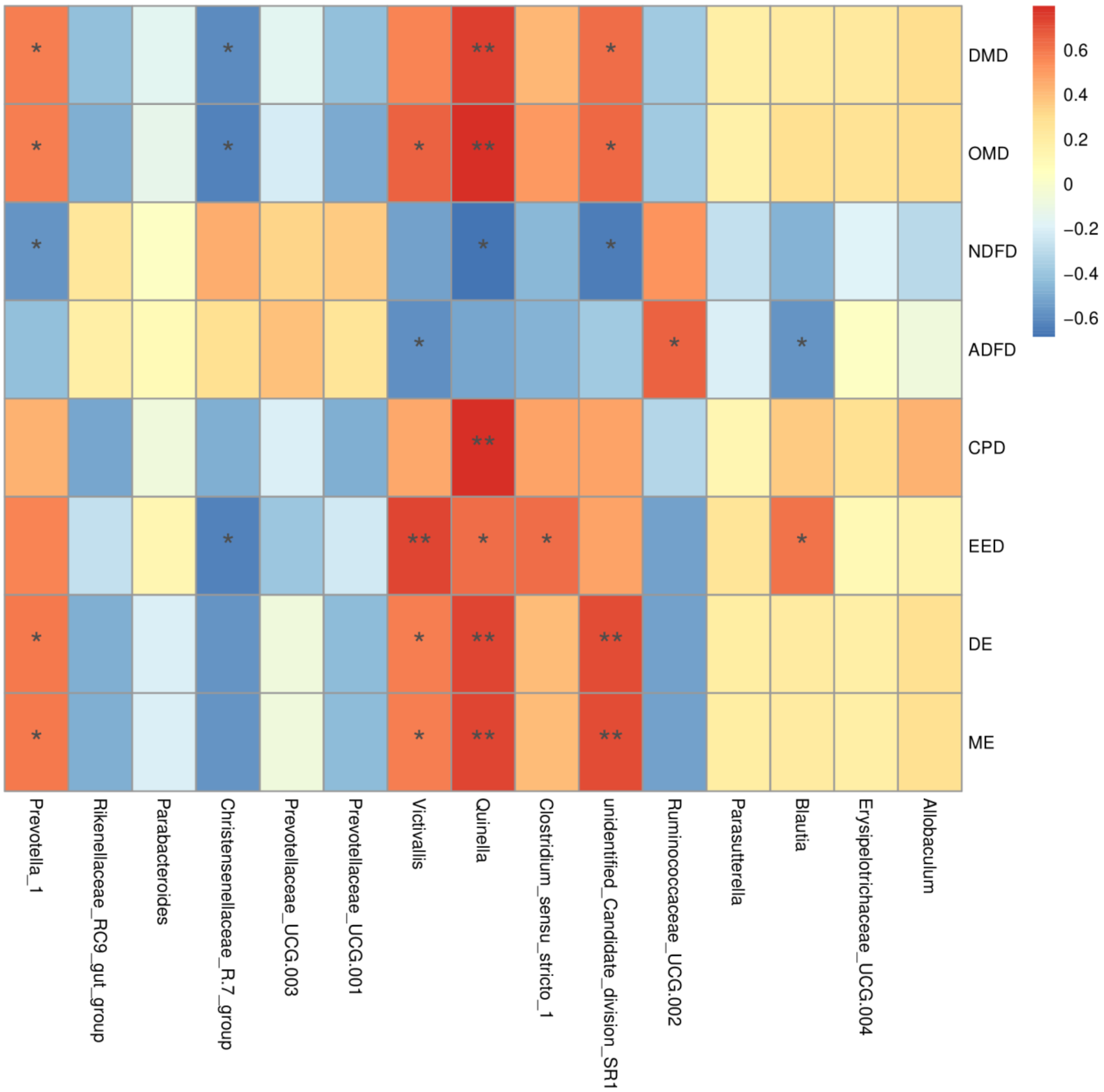

Supplementary Fig 4 Relationship among sheep' physiological status and bacterial genera (present at ⩾ 0.1% in at least one sample). Spearman' s rank correlation is shown as the color of the tile in this heatmap: Red means a positive correlation, blue means a negative correlation. * and ** indicate significance levels at 0.01 and 0.05, respectively. DMD, apparent digestibility of dry matter; OMD, apparent digestibility of organic matter NDFD, apparent digestibility of neutral detergent fiber; ADFD, apparent digestibility of acid detergent fiber; CPD, apparent digestibility of crude protein, DE, digestible energy; ME, metabolizable energy.
